# Modulation of cortical activity by spherical blur and its correlation with retinal defocus

**DOI:** 10.3389/fnins.2023.1184381

**Published:** 2023-07-13

**Authors:** Yannis Chenguiti, Samy Hamlaoui, Konogan Baranton, Satoru Otani, Elisa M. Tartaglia

**Affiliations:** ^1^Center of Innovation and Technologies Europe, Essilor International, SAS, Charenton-le-Pont, France; ^2^Sorbonne Université, INSERM, CNRS, Institut de la Vision, Paris, France

**Keywords:** sharpness of visual perception, EEG, spherical blur, retinal defocus, retinal image quality, eye model

## Abstract

Cortical activity, as recorded via electroencephalography, has been linked to the refractive error of an individual. It is however unclear which optical metric modulates this response. Here, we measured simultaneously the brain activity and the retinal defocus of a visual stimulus perceived through several values of spherical blur. We found that, contrary to the existing literature on the topic, the cortical response as a function of the overcorrections follows a sigmoidal shape rather than the classical bell shape, with the inflection point corresponding to the subjective refraction and to the stimulus being in focus on the retina. However, surprisingly, the amplitude of the cortical response does not seem to be a good indicator of how much the stimulus is in or out of focus on the retina. Nonetheless, the defocus is not equivalent to the retinal image quality, nor is an absolute predictor of the visual performance of an individual. Simulations of the retinal image quality seem to be a powerful tool to predict the modulation of the cortical response with the refractive error.

## Introduction

Since the discovery that visual stimulation induces specific cortical activation observable with electroencephalography (EEG), people have been interested in determining if specific patterns of cortical activity could provide a proxy for the quality of the visual perception and, eventually, could be used to prescribe the optimal correction to an individual.

In a clinical setting, the quality of the visual perception is defined by the visual acuity. When an individual subjectively reports poor visual perception and seeks medical advice, her/his visual acuity will be tested, and if the result is below normal capacity, her/his refraction will be measured and corrective lenses provided. Nonetheless, the refraction measurement is subjective in nature, relying solely on the patient's response; hence, its result can often be quite noisy and inaccurate. The patient himself is often unable to judge with certainty which correction gives the clearest image perception.

In this context, EEG is well-suited to provide a method for developing an objective refraction based on the recording of cortical activity [it is already well-known that measuring the visual evoked potentials -VEP- of the brain with EEG can be used for detecting and monitoring visual abnormalities (Simon et al., 2004; Naismith et al., 2009; Tello et al., 2010)]. As early as 1968, Harter and White (1968) looked at how the amplitude of the cortical response was modulated to a flickering checkerboard when placing various spherical lenses in front of the eye of a participant. They found that the amplitude of the response was maximal when the stimulus was viewed through the lens that corresponded to the subjective refraction. Since then, several studies have looked at the modulation of cortical activity using different paradigms (Millodot and Riggs, 1970; Duffy and Rengstorff, 1971; Ludlam and Meyers, 1972; Regan, 1973). All these studies led to the conclusion that the lens for which the highest cortical activity is elicited corresponds to the refraction determined subjectively, with a precision ranging from 0.25 to 0.5 dioptries. The amplitude of the cortical activity would then decrease symmetrically, for positive as well as negative lens values, relative to the maximum.

However, the question still lingers, what does the maximum of cortical activity signify? One could assume that it coincides with the best visual acuity, and hence with the sharpest perception at a given distance. However, the fact that there is one, and only one, maximum of cortical activity is hard to explain. Indeed, especially for young participants, when presented with lenses that require them to accommodate to see the stimulus, they should be able to easily do the effort, and thus have stable visual acuity (and hence a plateau of cortical activity) across a range of -negative- lens values (Millodot and Newton, 1981). On top of that, if the maximum of cortical activity indeed correlates with the subjective refraction, such a value would not necessarily coincide with the maximum of visual acuity. As a matter of fact, the subjective refraction measurement, if performed correctly, should limit visual acuity to minimize any symptoms of fatigue elicited by a sustained accommodation effort.

Alternatively, one can assume that the maximum of cortical activity is achieved when the stimulus is perfectly focused on the retina, and that cortical activity correlates to retinal focus. However, if this were the case, the decrease in cortical activity should not be symmetrical, it should decrease more slowly when the participant is wearing over myopic correction, as his accommodative response will partially compensate for the defocus. But since the retinal focus is never measured objectively, it seems hard to conclude that the variation of the amplitude of the cortical activity measured, corresponds to a variation the retinal focus.

Here, we propose, for the first time to our knowledge, to simultaneously record the cortical activity -with EEG- and the retinal focus, while placing different spherical lenses in front of a participant. This would allow us to track, simultaneously, the dynamics of the cortical response and of the retinal focus, in the effort to explore if and how recording the cortical activity could provide an objective method to identify the optimal correction.

## Materials and methods

### Participants

We tested 17 healthy volunteers, 10 females and 7 males, from 22 to 38 years old (mean age 26.06, std 4.44). Following the tenets of the Declaration of Helsinki, written informed consent was obtained from all participants after they were explained the goal of the study, as well as the test to be performed. None of the participants reported any known neurological or systemic disease, nor did take any medication that could influence the results of the test. All participants had normal or corrected to normal binocular visual acuity, determined through the subjective optimal refraction described hereafter.

### Stimuli

Stimuli were radial sine-wave gratings with blurred edges presented in a pattern-reversal mode, as typically done in SSVEP (steady state visual evoked potential) protocols (Norcia et al., 2015; Hamilton et al., 2021). The SSVEP refers to the brain response, observed via EEG, to a train of stimuli presented at a specific rate. This brain response oscillates at the same or a multiple frequency (harmonic) of the flickering frequency of the stimulus. Thus, the experimenter knows where to look in the signal to observe the hypothesized effect. The main advantages to the SSVEP method over the ERP (event related potential) method is the significantly shorter time of data recording needed to obtain a meaningful signal or observe the desired effect (i.e., a high signal to noise ratio). This method was first introduced using a flickering light (Adrian and Matthews, 1934), but much more complex images are used nowadays (faces, stereoscopic objects…). For most visual stimuli, the strongest signal is recorded from the electrodes located over the primary visual cortex, but it can be located elsewhere depending on the stimulus used. For example, when using faces, the strongest responses will be recorded from the electrodes placed over the right occipitotemporal cortex (Rossion and Boremanse, 2011). Also, the flickering frequency needs to be chosen carefully depending on the type of stimulus used, as it is going to influence at which frequency the highest response is elicited with the best *S*/*N* (Srinivasan et al., 2006). The general rule seems to be an inverse relationship between the flickering rate and the time needed to process the stimulus (Norcia et al., 2015), the more complex a stimulus is, the lower the flickering rate needs to be.

**Figure 1:**
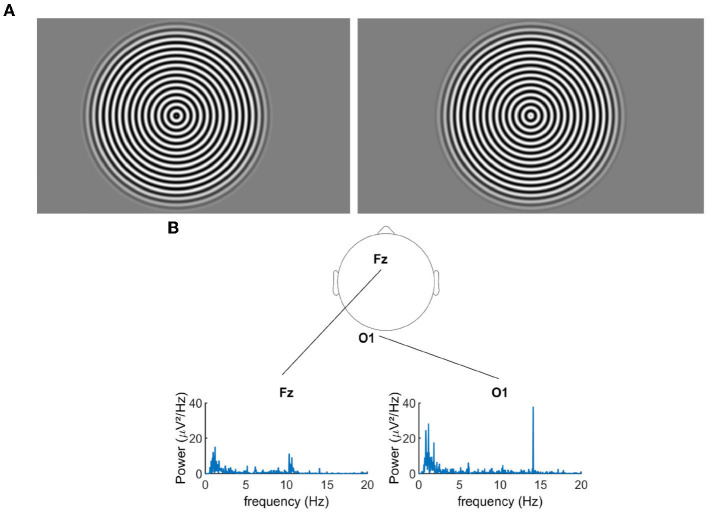
**(A)** Pattern reversal radial grating. **(B)** Example of the power spectral density for the signal recorded during the over-correction level “−0.75D" for two electrodes.

In the pattern-reversal presentation mode, our radial sine-wave gratings alternated between two states, where the white parts of the grating switched to the dark parts and vice-versa (see Figure 1A for an example). The advantage of this method is that both phases of the stimuli supposedly trigger equivalent neural populations and result in an EEG spectrum that contains only even harmonics (multiple of the flickering frequency of the stimuli).

The stimuli were generated using psychtoolbox via a Matlab script and displayed, on an Samsung OMH32 screen, at a rate of 7.04 Hz (corresponding to a duration of 142 ms) and comprising 14.08 reversal per second. The screen had a resolution of 1,920 by 1,080 pixel, a refresh rate of 125 Hz, and a mean luminance of 500 cd/m^2^. At 6 m, the stimulus patch subtended 3.8° of visual angle. The grating had a contrasts of 100% and a spatial frequency of 15 cpd. The luminance of the background was the average of the black and white part of the stimuli.

Note that the stimulus parameters (flickering frequency, spatial frequency as well as presentation duration) were optimized in terms of signal to noise ratio through a pilot experiment (data not shown). We also tested square wave stimuli to exclude any possible effect of blurred edges on the stimulus (see Supplementary Figure 1B).

### Procedure

The experiment took place in a dimly lit room, with the participant seated 6 m away from the display. Before starting the EEG recordings, the subjective optimal refraction (SOR), which corrects sphere and astigmatism, was determined as the maximum convex refraction that would allow the participant to have, binocularly, −0.1 logMAR of visual acuity at 6 m without any accommodation effort. If asked, an individual will most of the time subjectively prefer a slightly more myopic/less hyperopic refraction as it will increase her/his visual acuity. This refraction will however be less comfortable over time as the eye will permanently need to make an accommodative effort to maximize the visual acuity at far distance and an even more important effort at close distance.

The SOR was measured using the Vision R-800 (EssilorLuxotica, Paris, France), an automatic phoropter that offers continuous optical power changes by deforming several liquid lenses. This device allows for almost instantaneous lens power variation and it does not require to stack multiple lenses together to simulate various lens powers.

Participants were then equipped with the EEG cap; their visual perception was modulated by adding binocularly to the SOR a spherical lens power from +1 to −0.75 dioptries in 0.25 step (over-correction level). Note that the astigmatism was corrected for all participants. The cortical activity was recorded for 30 s for each over-correction level, in binocular conditions. The sequence of over-corrections was repeated in two consecutive trials. The EEG was synchronized with the Vision-R 800, each modification of lens by the Vision R-800 was marked in the EEG data at the precise time where the change occurred. Participants were asked to fixate a red dot placed at the center of the stimuli during the measurements and to move as little as possible, however they were allowed to blink as often as they wanted.

In the third trial, the retinal defocus (how much an image is out of focus from the retina) was measured using an open field autorefractor, the GrandSeiko WAM-5500 (Japan). EEG was recorded simultaneously. The sequence of over corrections tested was the same as before; however, lenses were placed on a trial frame and were changed manually each 30 s. The sequence of over-corrections was tested in a single trial.

### Data acquisition

EEG signals were recorded at a sampling rate of 500 Hz with a 32 channel electrodes system (EEGO sports, ANTneuro). The electrodes placement corresponds to the 10–20 standard system. The impedance of each electrodes were kept below 20 kOhm during the entire recording. The data were recorded using a custom Matlab program that was also retrieving the trigger sent by the Vision R-800 corresponding to the change of lenses. The retinal defocus was recorded with a GrandSeiko WAM-5500 at a rate of 6 Hz.

### Data pre-processing

The retinal defocus data were cleaned by applying a moving window of 5 s to the data. Data points that were more than three standard deviations off the median of the windows were defined as outliers and removed. Data for three participants were excluded, since the device was unable to acquire the data in a reliable way.

EEG data were filtered between 1 and 40 Hz and re-referenced to the average of the 32 electrodes. From the 30 s recorded, the length of all epochs was set to be the highest multiple of 71 sample points (corresponding to 142 ms at 500 Hz and a frequency of 7.04 Hz). This was done to precisely estimate the power of the signal at our frequency of interest when performing the Fourier transform. A Fast Fourier Transform was applied to each epoch. Only the real part of the output, i.e., the magnitude, was kept and squared to compute the power of the signal. The resulting EEG power at 14.08 Hz, i.e., the second harmonics of the flickering frequency of the stimuli, was normalized between 0 and 1, for each participant, and taken as the index of the effect of spherical blur.

### Fitting procedure of EEG data

We implemented a model that, given the cortical response to the flickering stimulus perceived through the tested sequence of over-corrections, identifies the lens that corresponds to the SOR. If the model is correct, we should be able to retrieve the SOR for each participant from her/his EEG signal, with sufficiently high accuracy.

To do so, we extracted from the EEG signal an estimation of the spectrum using the Welch periodogram method. This method splits the entire signal into windows of specified durations, estimates the spectrum for each window, and averages them together. The Welch method has the advantage of reducing the noise in the signal by sacrificing frequency resolution. This loss of resolution is not damaging to us as we are only interested in very specific frequencies, i.e., the flickering frequency of the stimulus and its harmonics. We applied Welch on the following channels: O1, Oz, O2, Poz, P3, Pz, P4 and, for estimating the stimulus harmonics from the spectrum, between 0 and 40 Hz. We ended up with a plot of the peaks of cortical activity as a function of the tested over-corrections. Next, we set out to fit a sigmoidal function to this data. Not all the over-correction values were considered at once for the fitting, but rather a subset of five or seven consecutive values. This resulted in six sigmoids fitted for each channel and frequency. For all sigmoids, its RMSE was calculated. We kept the 5% for which the RMSE was the highest and made the prediction that the over-correction level corresponding to the inflection point of the fitted sigmoid was the subjective refraction (SOR).

The prediction for each sigmoid was then aggregated to create a probability distribution for which the over-correction was the most susceptible to correspond to the SOR.

The final decision was then conducted either by considering the correction that had the highest probability or the center of mass of the probability distribution.

### Eye model

An optical model (Atchison, 2006) was used to simulate the propagation of light through the different ocular surfaces, until the light hits the retina, with the aim to predict and quantify the retinal image quality of our stimuli (Marcos, 2003). More in details, the model allows us to take into consideration the role of pupil size, diffraction and high order aberrations on the retinal image quality of the normal human eye. Importantly, the model features some anatomical characteristics of the eye such as aspherical optical surfaces and a gradient-index crystalline lens. This allows for a good representation of average higher order aberrations of the eye in an adult population. For instance, the primary spherical aberration (Zernike coefficient $C_{4}^{0}$) for the emmetropic version of the model is about 0.12μm for a 6 mm pupil size, which is close to the population average Value (0.13μm as reported by Salmon and van de Pol, 2006).

Here, we used the model to calculate through-focus retinal image quality for various pupil sizes, in order to assess the impact of spherical aberration on the best-focus position. The modulation transfer function (MTF) is one common method to measure the optical performance of an imaging system and therefore the image quality (Goodman, 2005; Born and Wolf, 2013). Hence, the MTF can be applied to the human eye in order to assess the retinal image quality. More specifically, the MTF is the modulus of the normalized Fourier transform of the eye point spread function (PSF), i.e., the image of an object point through the eye. The MTF represents the decrease in sinusoidal grating contrast as a function of spatial frequency, and we can expect the MTF value at a frequency corresponding to the stimuli of interest (15 cpd grating in our case), to be a reasonable estimator of the retinal image quality. Note that, the MTF of the retinal image calculated from the model (for an emmetropic observer), is a fairly good estimator of that measured in human subjects with double pass methods (Westheimer and Campbell, 1962; Berny, 1969; Charman and Jennings, 1976; Santamaría et al., 1987; Artal et al., 1995; Liang and Williams, 1997).

However, one obvious limitation is that the MTF does not take into consideration further cortical levels of visual information processing. Such limitation can be accounted for by using the contrast sensitivity function (CSF, the change of contrast perception as a function of spatial frequency) as a weighting function for the MTF. Therefore, an additional way of estimating the image quality of a grating objects while accounting for both optical aberrations and psychophysical specificities, is to compute the VSMTF (), i.e., the visual Strehl ratio computed in the frequency domain, where the MTF is weighted by the neural contrast sensitivity function *CSF*_*N*_:


(1) $$VSMTF=\frac{\int_{{\mathbb{R}}^{2}}CSF_{N}(f_{x},f_{y})MTF(f_{x},f_{y})df_{x}df_{y}}{\int_{{\mathbb{R}}^{2}}CSF_{N}(f_{x},f_{y})MTF_{DL}(f_{x},f_{y})df_{x}df_{y}}$$

where, *MTF*_*DL*_ represents the MTF of a diffraction limited imaging system for the relevant pupil size. Various *CSF*_*N*_ exist in the literature, and a common one, which we used, has been described in Campbell and Green (1965). Moreover, phase transformations contribute to the retinal image quality and may be assessed, along with the contrast modulation, by using a different metric, the VSOTF:


(2) $$VSOTF=\frac{ℜ\int_{{\mathbb{R}}^{2}}CSF_{N}(f_{x},f_{y})OTF(f_{x},f_{y})df_{x}df_{y}}{\int_{{\mathbb{R}}^{2}}CSF_{N}(f_{x},f_{y})OTF_{DL}(f_{x},f_{y})df_{x}df_{y}}$$

where


(3) $$OTF(f_{x},f_{y})=\frac{ℱ\{\text{PSF}(x,y)\}(f_{x},f_{y})}{ℱ\{\text{PSF}(x,y)\}(0,0)}$$

and *OTF*_*DL*_ is the *OTF* limited by the diffraction. Note that the MTF is the modulus of the OTF, therefore the information about the phase is lost. The VSOTF provides a rigorous way to quantify the retinal image quality.

## Results

**Figure 2:**
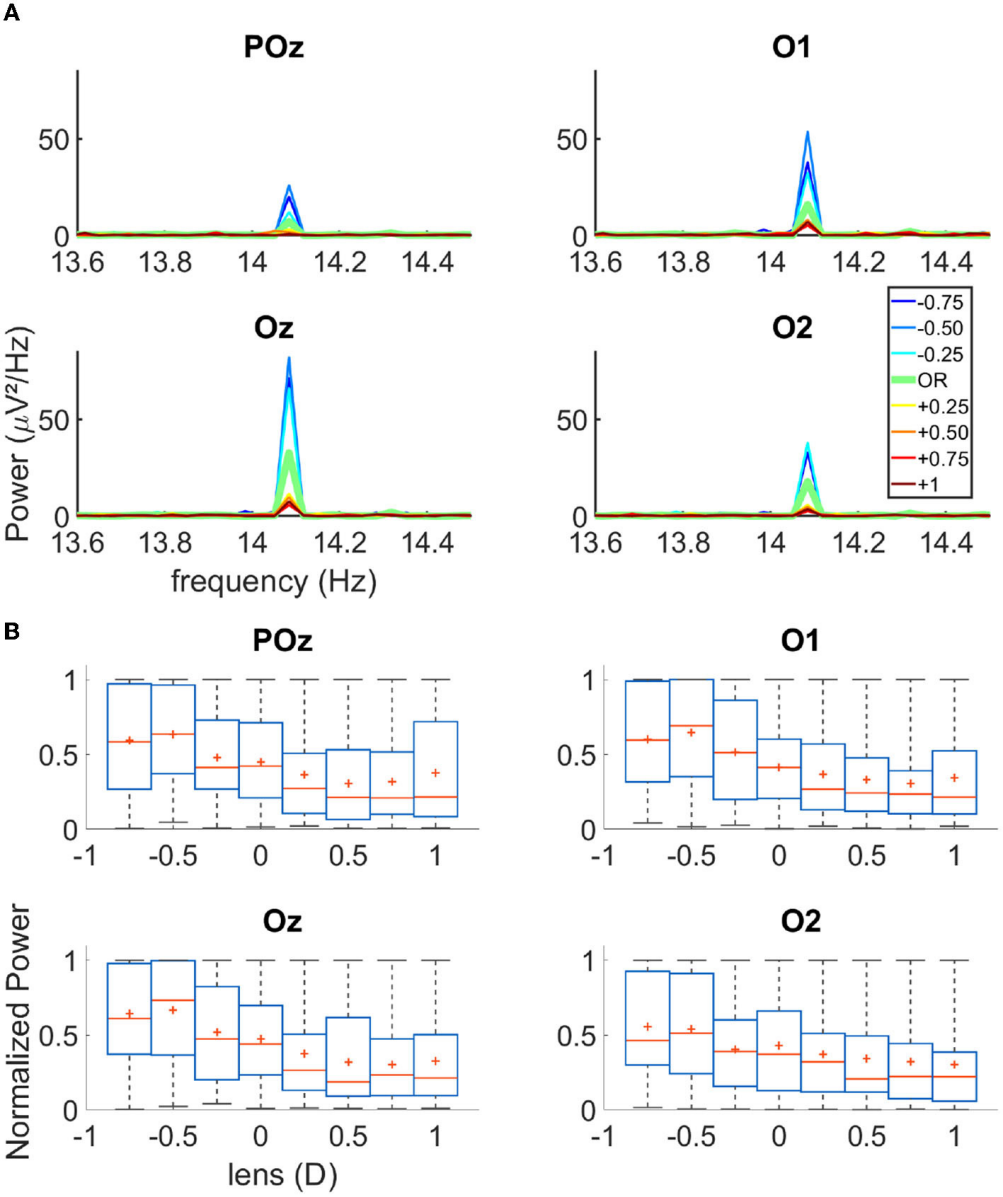
**(A)** Spectrograms of the activity recorded on four occipital channels for one single subject. **(B)** Boxplot of the normalized power for each lens condition of all trials for all participants *N* = 17; The red cross represents the mean of the data, the red line the median, the blue box the first and third quartile, and the gray bar the min and max value.

First, via the EEG data analysis, we verified that the flickering gratings indeed elicited a significant cortical signal in the occipital cortex, with an acceptable signal to noise ratio. Figure 1B shows the power spectral density of the EEG signal recorded from electrodes Fz and O1 (for an exemplary over-correction level of −0.75D). We observed an important peak of activity at 14.08 Hz for O1, which is the second harmonics of the flickering frequency of our stimulus, in accordance with the pattern reversal nature of our stimuli (Norcia et al., 2015). We had the highest response over the occipital region for the electrodes POz, O1, Oz, and O2. Note that in the frontal lobe, the activity at 14.08 Hz was almost non-existent, as expected for this kind of visual stimulation. Figure 2A shows the cortical activity measured for the electrodes POz, O1, Oz, and O2 for a single participant, for different over-correction levels. We can clearly observe a trend, for all 4four electrodes. The positive over-correction level elicits the lowest activity and the negative over-correction level elicits the highest activity (notably the “−0.5D" level).

Next, we plot the normalized power of the cortical signal for the three trials of all participants (*N* = 17) as a function of the lens power (Figure 2B). We observed that both the mean and median cortical response increase from +1D to −0.75D. The maximum response occurs either at “−0.75D" or “−0.50D" depending on the electrodes. This is not in agreement with the literature, which rather states that the refraction of a participant, the “0D" condition, elicits the highest activity; in our case, an over myopic correction produces more power in the cortical response. Furthermore, we observed that the cortical activity as a function of the over correction levels had a sigmoidal rather than a bell-shaped behavior, as previously reported in the literature. When fitting a sigmoidal function over the data (see the fitting procedure in the Method section), we could indeed retrieve the SOR from the inflection point of the sigmoid. The accuracy of the model was close to 100% when we allowed 0.25D of tolerance on the SOR (see Supplementary Figure 1B).

**Figure 3:**
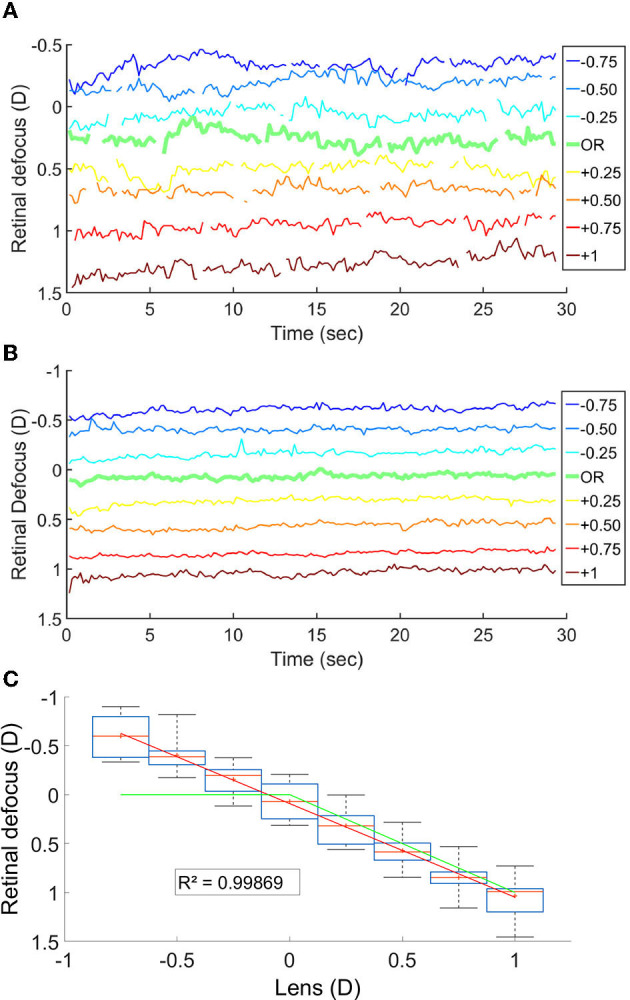
**(A)** Retinal defocus in time for a participant. **(B)** Mean retinal defocus in time for all participant *N* = 14. **(C)** Boxplot of the mean retinal defocus for each lens condition *N* = 14; The red line is the median, the red cross is the mean, the box indicate the first and third quartile and the bar the min and max value. The green line represent the defocus in a theoretical system where the accommodation response focus the stimuli onto the retina.

We measured the retinal defocus simultaneously to the EEG recordings, while changing lens power in front of the participant. The goal was to verify whether cortical activity peaked when the stimulus was perfectly on focus on the retina. Figures 3A–C shows the variation in time of the retinal defocus for one participant, as well as the mean for all participants. Figure 3C shows the mean and median over the 30s of stimulus presentation of the retinal defocus for all participants as a function of over-correction level.

**Figure 4:**
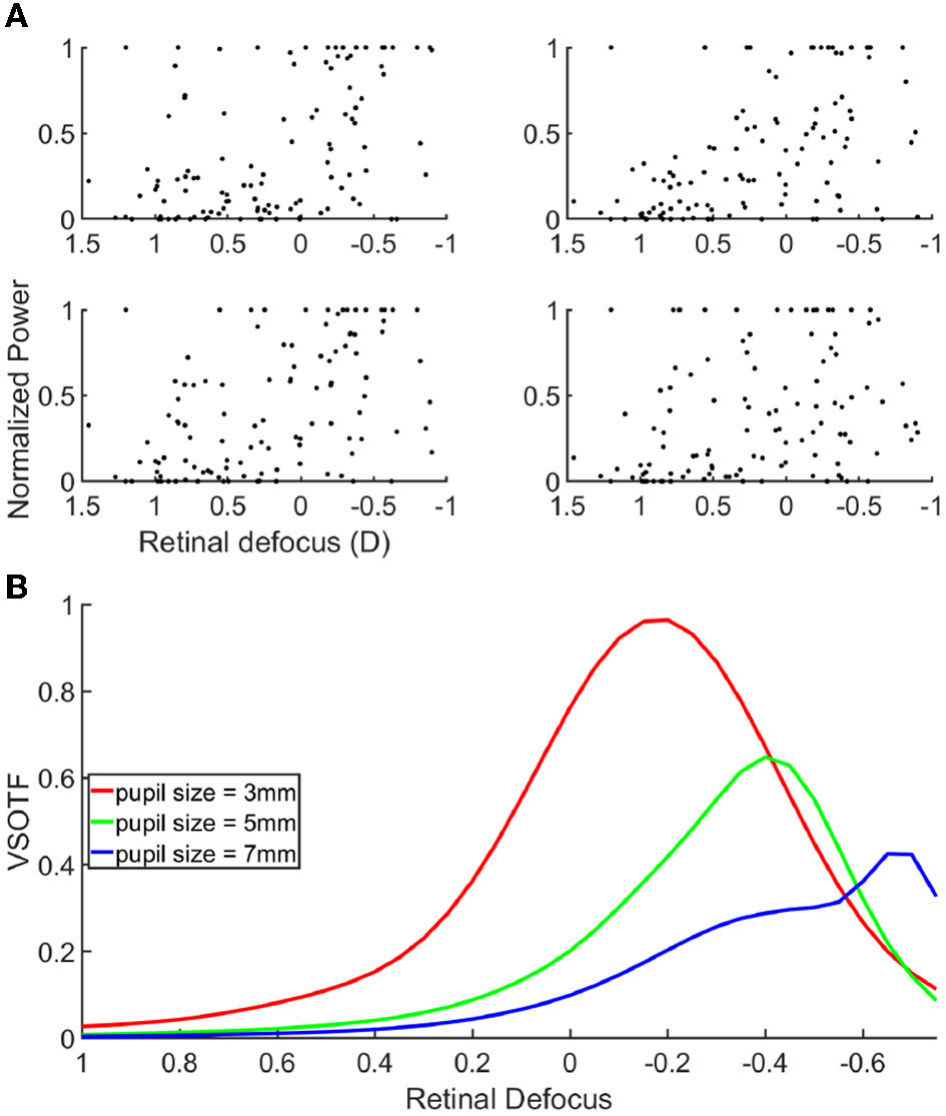
**(A)** Scatter plot of the cortical response as a function of the retinal defocus. **(B)** Contrast gain through a simulated eye, for a spatial frequency of 15 cpd, as a function of defocus. Each curves correspond to a different pupil size, 3 mm = red, 5 mm = green, 7 mm = blue.

The fact that the mean retinal defocus is almost 0 for the over correction level “0D" indicated that indeed our participants were properly corrected, and that the subjective correction we find is adequate. However, most importantly, we observed a linear relationship between the lens added to the SOR and the retinal defocus (R-squared: 0.999), indicating an average accommodative effort almost null across all lens conditions. Consequently, overcorrection values and defocus mainly coincide. We observed no linear correlation when plotting the scatter plot of the power of the cortical response as a function of the retinal defocus (Figure 4A).

Next, we set out to analyze if and how the cortical activity would rather correlate with the retinal image quality, than with the retinal defocus. If verified, this hypothesis would signify that the cortical activity is modulated by a combination of several optical parameters, like, for example, the pupil size, and the higher order aberrations of the eye. To validate our hypothesis, we used an emmetropic eye model to simulate and quantify the retinal image quality of our stimuli. Figure 4B shows the VSOTF of an average emmetropic eye, as a function of the defocus from the retina. The VSOTF represents a metric to quantify the retinal image quality, and it has the advantage of accounting for important psychophysical features; the lower the VSOTF value, the worse the image quality. We found a very good agreement between the VSOTF and the MTF at 15 cpd (see Supplementary Figures 2A–C). The MTF describes the stimulus contrast gain at the retina level; the lower the MTF value, the lower the stimulus contrast gain. Hence, one can interpret the behavior of the curves in Figure 4B, equivalently, in terms of retinal image quality and stimulus contrast gain. The three curves indicate the VSOTF for different pupil diameters, when other optical parameters have been set to known averaged population values. Several observations can be made from this figure. First of all, the image quality is not maximal when the stimulus is on focus onto the retina, illustrating that retinal defocus is indeed not equal to retinal image quality. Moreover, we can observe that the image quality increases with the decrease of the pupil diameter, which is a known effect (Schwiegerling, 2000). Like the defocus, optical aberrations blur the retinal image, reducing image contrast and limiting the range of spatial frequencies available to further stages of visual processing. The contribution of aberrations to optical degradation is typically smaller than that of defocus or astigmatism. However, the blurring effect of aberrations becomes more noticeable for larger pupils. We can also observe a shift in the relative maximal contrast gain as the pupil size changes. The more the pupil size increases, the more the maximal contrast gain shifts toward negative defocus values (i.e., the stimulus is focused behind the retina). Hence, the retinal image quality improves for negative over-correction values. These results could explain why we find that the cortical response is maximal for negative over-correction values, rather than for the SOR value, or analogously, rather than for an optimal retinal focus. In other words, the retinal image of the stimuli is more contrasted for negative over correction values, and thus elicits a higher cortical response. In accordance with our hypothesis, when looking at the correlation between the cortical activity and the VSOTF (see Supplementary Figure 3), we do find that the correlation increases with pupil size, and it is close to 1 for a 7 mm pupil. Our subjects had a pupil diameter that ranged between 3 and 7 mm (see Supplementary Figures 4A, B); however, the measure of the pupil diameter provided by the Grand Seiko was very unstable, and hence not very reliable, because of the reflection on the worn lenses.

## Discussion

We recorded cortical activity via EEG while modulating the sharpness of a flickering visual stimulus through negative and positive corrective lenses; simultaneously, we measured the retinal defocus of the stimulus via an open field autorefractor, under the hypothesis that the cortical activity might correlate with it. We found that the cortical activity correlates with the retinal image quality, rather than the defocus of the stimulus on the retina, and hence might potentially be affected by a combination of several other optical factors.

The idea to look at cortical activity to provide an objective measure of the optimal correction exists since a long time (Harter and White, 1968; Millodot and Riggs, 1970; Duffy and Rengstorff, 1971; Ludlam and Meyers, 1972; Regan, 1973). Most of these studies, however, fail to clarify what is that one measures -in the brain- when modulating the sharpness of visual perception by means of corrective lenses. One potential source of confusion comes from the observation that the cortical activity peaks at the subjective optimal correction (SOR), i.e., at the lens value found via subjective refraction, but no detail is given on how such subjective refraction is measured (Harter and White, 1968; Millodot and Riggs, 1970; Duffy and Rengstorff, 1971; Ludlam and Meyers, 1972; Regan, 1973). Hence, it remains unclear whether, in the process of determining the SOR, visual acuity was maximized or rather limited to minimize accommodation. In our case, the SOR was determined to privilege minimum accommodation effort over maximal visual acuity. This is likely the reason why we found that the highest amplitude of cortical activity did not correspond to the SOR, but rather to an over myopic correction (of approximately −0.5D). However, it should be noticed that this result can also be related to the tendency of subjects to more easily accept on the short term a slight under-hyperopic or over-myopic correction [e.g., 50% of population wearing glasses in India have a refractive error of 0.5D (Sheeladevi et al., 2019)].

Moreover, importantly, instead of the typical bell-shape with a unique maximum and a symmetrical decrease around it (as reported on a large part of the classical literature cited above) the cortical response that we recorded follows a sigmoidal behavior, with the maximum of activity spreading across a plateau of negative lens values, and the SOR coinciding with the inflection point of the sigmoid. The easiest explanation of such an asymmetry could have been that our participants, contrary to those in the above-mentioned studies, do deploy an accommodative effort in order to keep the stimulus in focus when wearing negative lenses.

However, unexpectedly, we observed from the open field autorefractor data, recorded simultaneously to the EEG signal, a linear relationship between the overcorrections and the retinal defocus, indicating the absence of an accommodative effort across all lens conditions. In a theoretical system, for negative lenses, the accommodation response would have nullified the defocus (green curve in Figure 3C).

Nonetheless, the accommodation response does not only depend on optical factors. More high-level, cognitive processes could affect the accommodation response. For example, the accommodation response is higher for high attention-demanding tasks compare to passive tasks (Francis et al., 2003). Thus, in our case, the lack of an active task could explain the lack of accommodation measured. Additionally, it has been suggested that the role of the accommodation response is not necessarily to maximize the contrast, but to achieve a “good enough” visual performance (Bernal-Molina et al., 2014). This balance, between the accommodation response and the visual performance, will depend, as mentioned above, on the visual task but also on the stimulus. Indeed, The lower the spatial frequency content of the visual stimulus, the lower the accommodation response will be, since a greater defocus does not reduce visual performance. Thus, in our case, the use of a fairly low spatial frequency of 15 cpd would partially explain the lack of accommodative response for the negative lens. Lastly, the accommodation could have been hindered by the flickering of the stimulus.

Even more surprisingly, we found that the amplitude of the EEG response does not correlate with the absolute value of the retinal defocus (Figure 4A). Thus, cortical activity is not a good predictor of retinal defocus. If it was the case we would have had a maximum amplitude of the cortical activity for the “0D" condition, where the retinal defocus is minimal. Furthermore, the signal would have decreased symmetrically for positive and negative retinal defocus values. Instead, the behavior of the cortical activity rather correlates with the theoretical retinal defocus, which would be null when the accommodative response compensates the defocus induced by the negative lenses (see green curve in Figure 3C). Then, how to reconciliate the behavior of the cortical activity knowing that our measurements did not show any compensating accommodation response for negative lens values? We put forward the hypothesis that the cortical activity correlates with the retinal image quality rather than with the retinal defocus. This implies that retinal image quality and retinal defocus do not coincide. As a matter of fact, the former is defined by a significant amount of other factors such as optical aberrations, pupil diameter, light intensity, eye transparency etc. Notably, a symmetric defocus around the retina, in the presence of a certain amount of spherical aberrations, will correspond to dissimilar contrast transfers for specific spatial frequencies, hence yielding asymmetric retinal image quality. This has been shown by simulations run on an emmetropic eye model, which takes into consideration all the above mentioned optical factors. The simulation results show that at a spatial frequency of 15 cpd, i.e., the spatial frequency of the flickering stimulus we used, the contrast gain will be higher in the −0.5*D* condition compared to the +0.5*D* and will decrease more slowly for negative over-correction values, yielding a better image quality. As a result, we do find an analogous behavior in the cortical activity, with higher peak amplitudes for negative lens values. Note that, in the model, the observed effect of contrast gain increases with the pupil diameter. We chose to study contrast gain as a function of the pupil diameter, while using a fixed, averaged value for the spherical aberrations. Although the two parameters are expected to be correlated, in the future it might be interesting to disentangle the two by measuring spherical aberrations individually for each participant, and use those values to run the model simulations.

It has been shown (Webster et al., 2002; Webster, 2015) that the perceptual judgement of blur is biased after blur adaptation. Hence, one could expect such an adaptation to be reflected on the cortical activity. However, we found that the amplitude of the EEG signal does not decrease significantly over the 30 s of stimulus presentation, for any of the over-correction tested (see Supplementary Figure 5). Moreover, the accuracy on the estimation of the inflection point of the sigmoid remains rather constant across the 30 s of stimulus presentation (see Supplementary Figure 1A). If the signal was affected by adaptation, its decrease over time would have entailed a decrease in the accuracy as well.

As stated before, contrary to most of the existing literature on the subject, we found that the lens condition corresponding to the SOR does not elicit the highest response and that an under-correction of the hyperopia or an over-correction of the myopia elicits a higher power. It seems that how the SOR as well as visual acuity are measured in those studies might be an issue and should be managed case by case. To this regard, it is important to stress that visual acuity, as measured classically in a refraction exam, is not a good metric to investigate the relationship between clearness of perception and cortical activity. In itself, visual acuity indicates the highest spatial frequency an individual is able to discriminate. The classical stimuli used to measure visual acuity, i.e., the optotypes, have varying spatial frequencies and sizes and, hence, each will potentially elicit a different modulation of cortical activity. Spatial frequency and size of the stimulus, simply put the stimulus changing parameters, will then become confounding variables when searching for the effect of the clearness of the perception on cortical activity. In our case, the stimulus used has a fixed size and a fixed spatial frequency, providing a way to work around this problem. In future works, it would be interesting to combine our stimulus with a gradation metric approach (Legras and Benard, 2013) in which participants are asked to grade from 0 to 100 (0 being bad and 100 being excellent) the clearness of their visual perception while viewing a stimuli with fixed parameters through different lenses. This method would allow to assess if the gradation score, i.e., a subjective proxy of the clearness of the visual perception, correlates with the amplitude of the cortical activity.

## Data availability statement

The raw data supporting the conclusions of this article will be made available by the authors, without undue reservation.

## Ethics statement

The studies involving human participants were reviewed and approved by Comite de Protection des Personnes Ile de France III Hopital Tarnier-Cochin, Paris. Email: cpp.iledefrance3@orange.fr. The patients/participants provided their written informed consent to participate in this study.

## Author contributions

YC, ET, and SO contributed to conception and design of the study. YC recorded the data and performed the analysis. YC and ET wrote the first draft of the manuscript. SH and KB wrote sections of the manuscript and performed the model simulations. All authors contributed to manuscript revision, read, and approved the submitted version.
