# Supplementary material for: Modulation of cortical activity by spherical blur and its correlation with retinal defocus
Source: Front Neurosci. 2023 Jul 13;17:1184381. doi: 10.3389/fnins.2023.1184381 (PMC10372438; doi:10.3389/fnins.2023.1184381)
Supplement: Supplementary file 1 [file Table_1.pdf]

Appendix

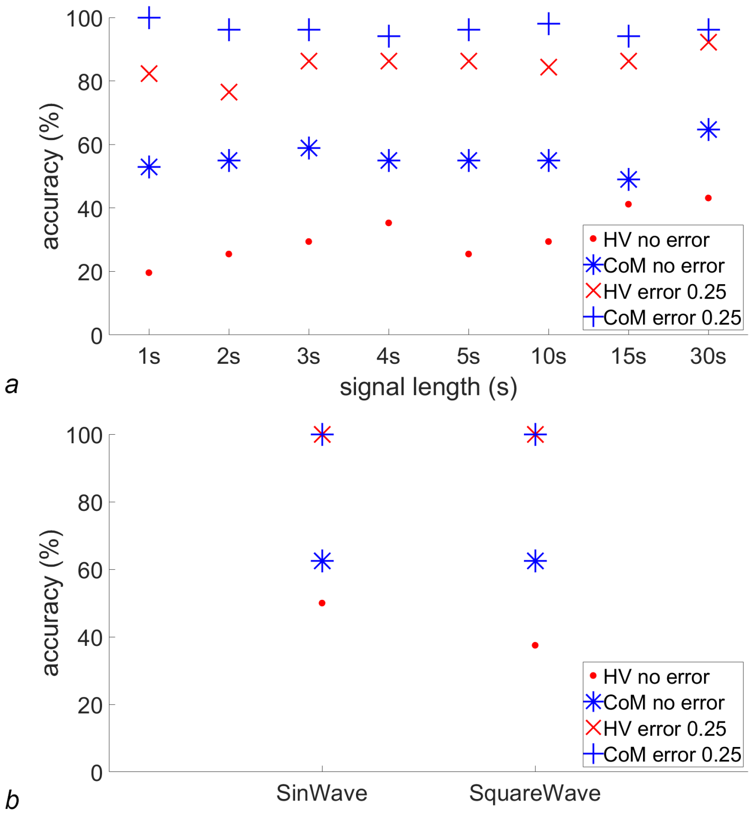

Figure 1: a: Model accuracy for the final decision on the SOR; signal length indicates the length in seconds of the signal segment considered for the analysis. HV indicates that the decision on the SOR was made in favour of the correction that had the highest probability (red dot: the final decision corresponds exactly to the SOR; red cross: the final decision corresponds to the SOR ± 0.25D). CoM indicates that the decision on the SOR was made in favour of the center of mass of the probability distribution (blue star: the final decision corresponds exactly to the SOR; blue cross: the final decision corresponds to the SOR ± 0.25)/ b: Accuracy for the final decision on the SOR depending on the luminance profile of the stimulus: sin-wave or square-wave.

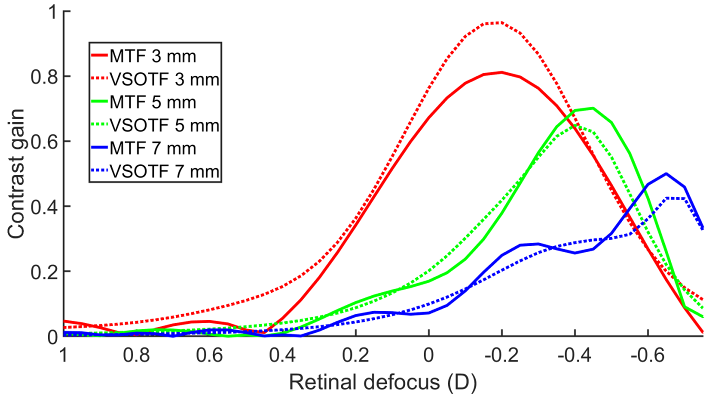

Figure 2: Comparison between the MTF and the VSOTF for different pupil sizes. The MTF is shown in solid line, the VSOTF is shown is dotted line

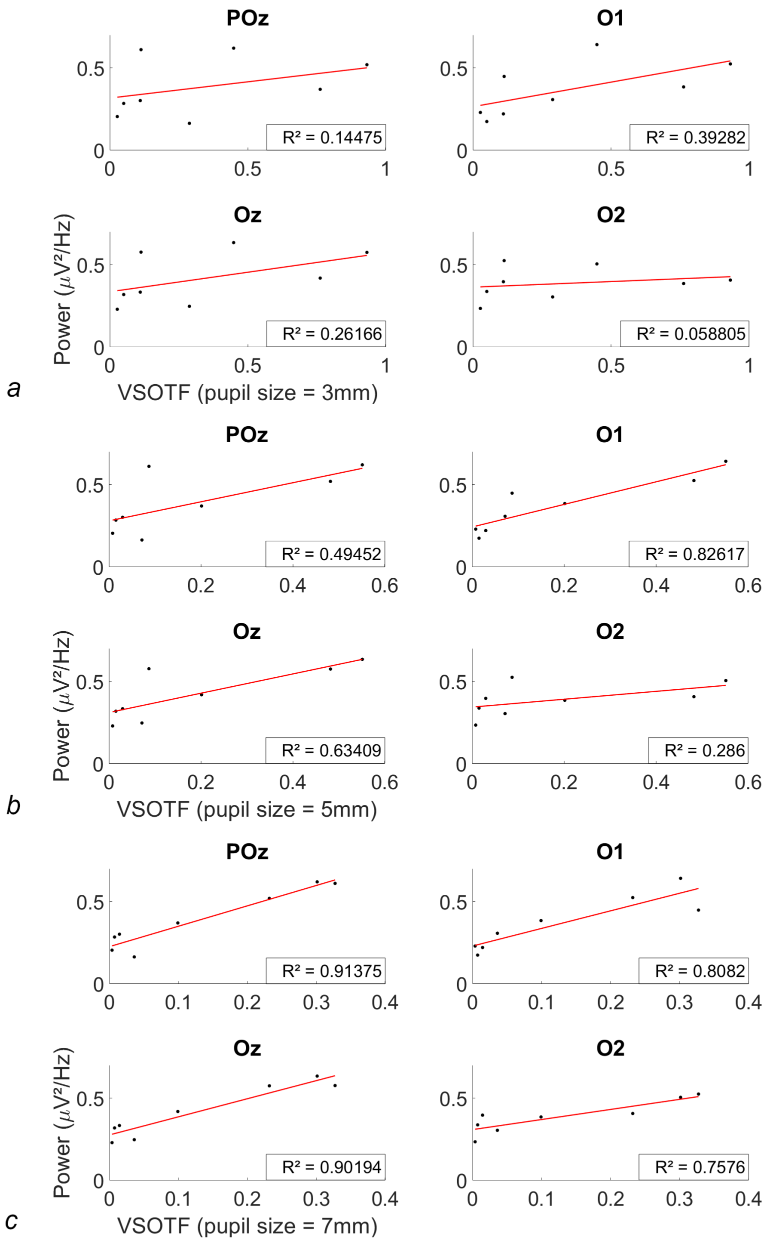

Figure 3: Linear regression between the mean normalized power of the EEG signal over the four main electrodes, and the VSOTF, for the same retinal defocus value. / The pupil size is set at: a: 3mm; b: 5mm; c: 7mm. The correlation between cortical activity and retinal image quality increases with the pupil diameter.

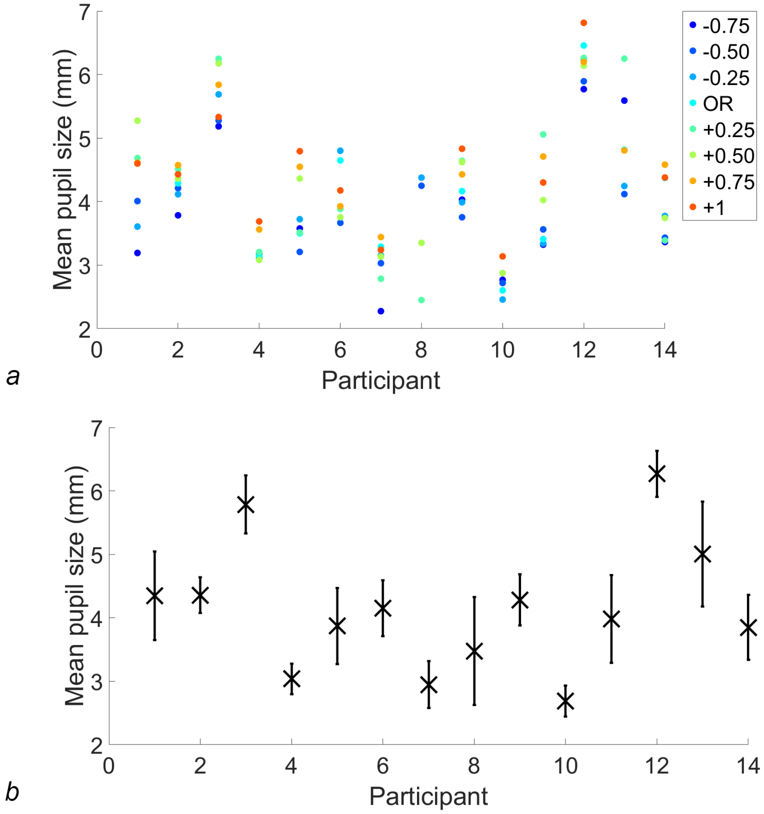

Figure 4: a: Individual pupil size, measured by the GrandSeiko, averaged over 30s of presentation for each overcorrection level. Some data points are missing due to the inability of the device to accurately track the pupil through the different lenses/ b: Individual pupil size, averaged over 30s of presentation and over overcorrection level.

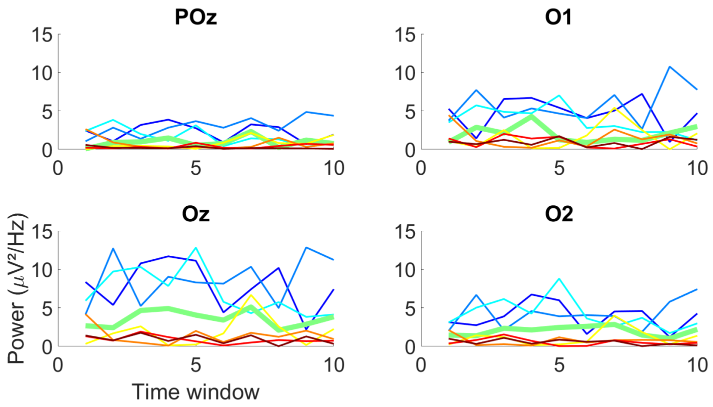

Figure 5: Example for a single subject of the cortical activity at the frequency of interest depending on the overcorrection level and a sliding windows of 3s over the 30s presentation. Each point corresponds to the FFT at 14Hz computed over 3s of the signal. To investigate a possible neural adaptation to blur over the 30s of each overcorrection level, we split, for each participants, the signal into 10 segments of 3s and computed the FFT to retrieve for each segment and each overcorrection level the cortical activity at 14Hz. We then calculated the linear regression, for a given subject/overcorrection, of the cortical activity as a function of the time segment. If a neural adaptation occurred, and that for example, the cortical activity was consistently lower at the end of the 30s of presentation time, we would find on average a negative coefficient for the linear regression. In our case, when averaging all participants, occipital channel, and overcorrection levels, we find a coefficient of 0.0002 with a standard deviation of 0.08. This seems to indicate that no neural adaptation occurs during our recordings.
