# Supplementary figures and images for: Modulation of cortical activity by spherical blur and its correlation with retinal defocus
Source: Front Neurosci. 2023 Jul 13;17:1184381. doi: 10.3389/fnins.2023.1184381 (PMC10372438; doi:10.3389/fnins.2023.1184381)

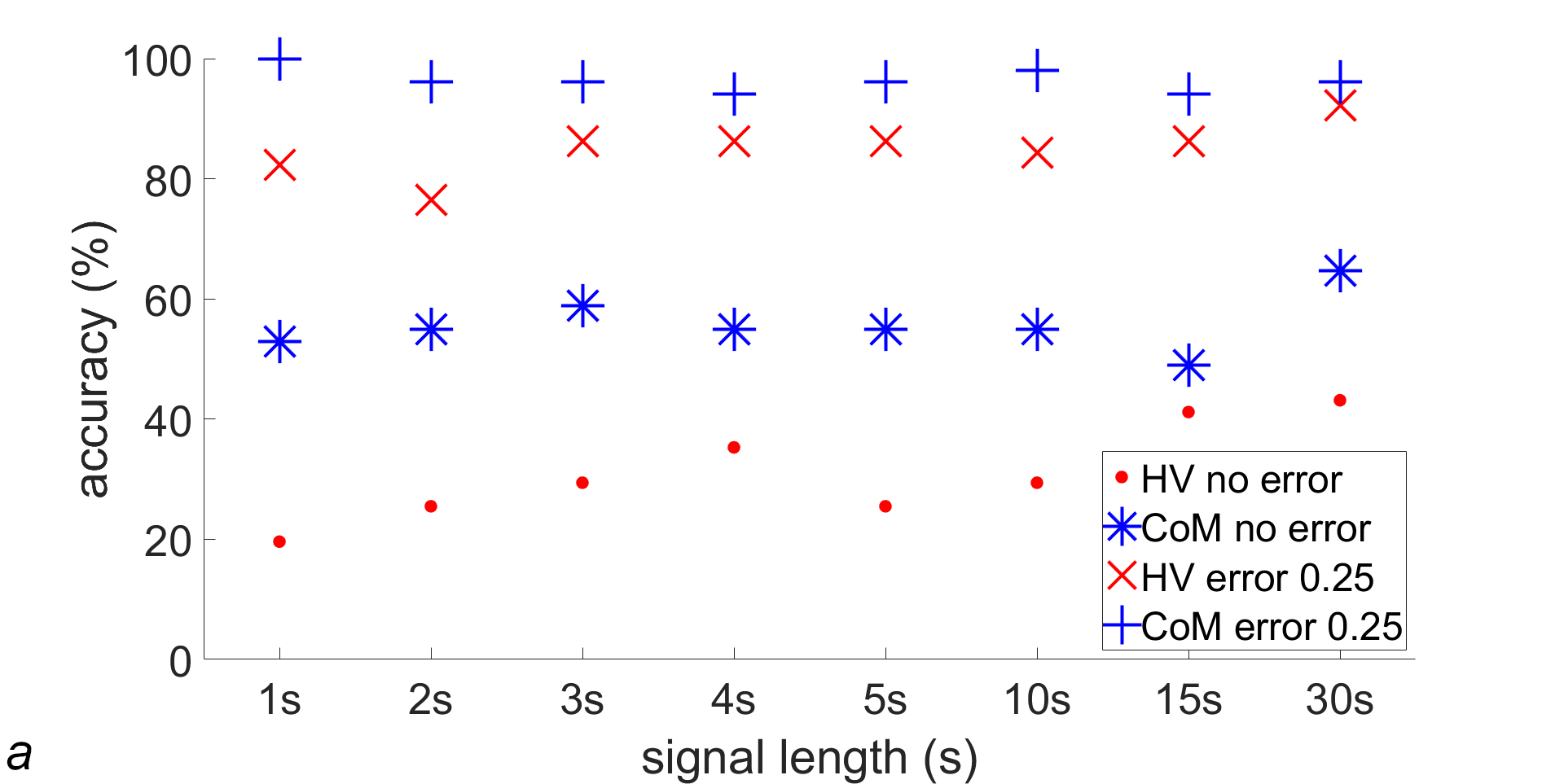

Supplement: Supplementary file 2 [file Image_1.tif]

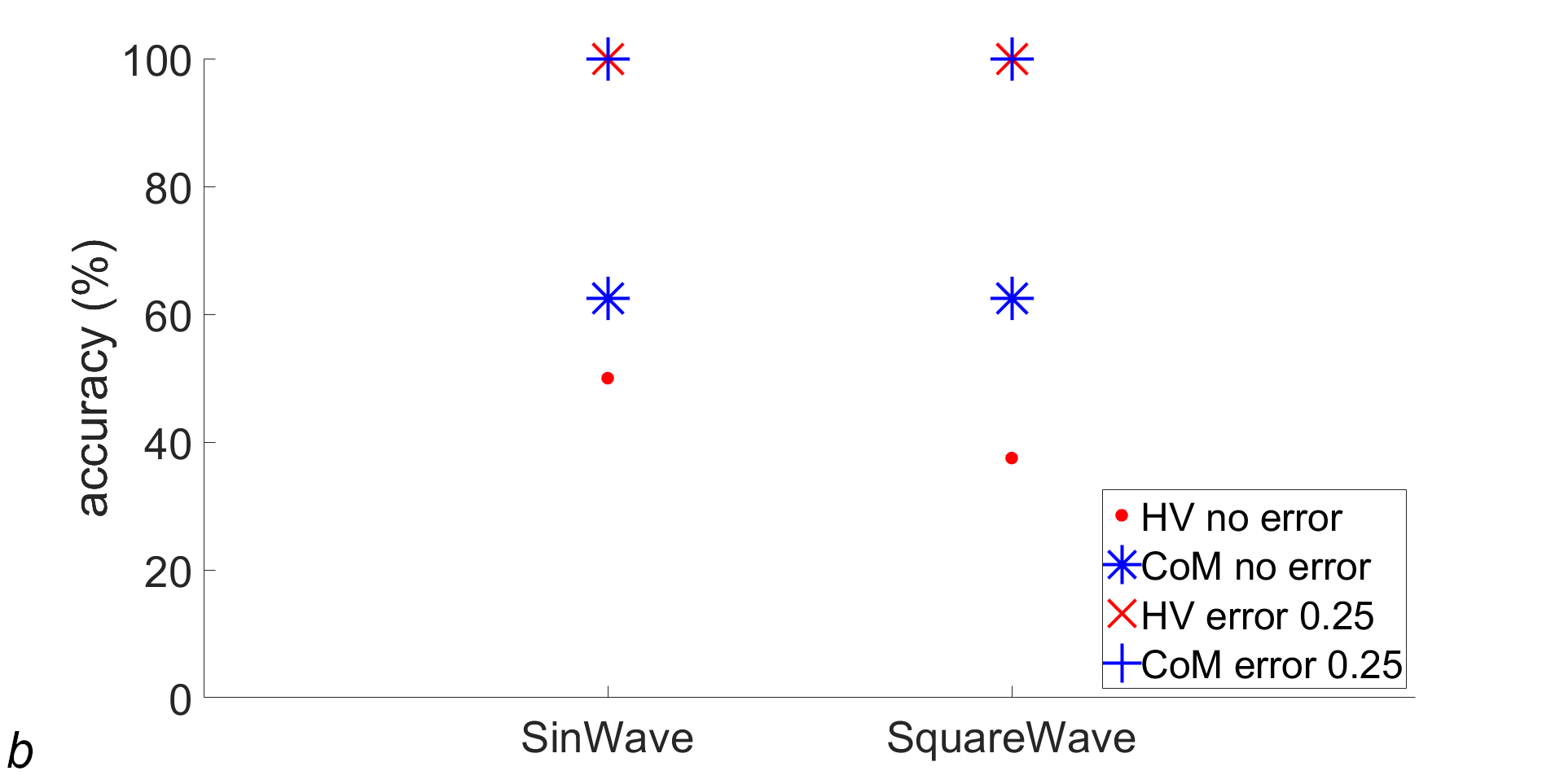

Supplement: Supplementary file 3 [file Image_2.tif]

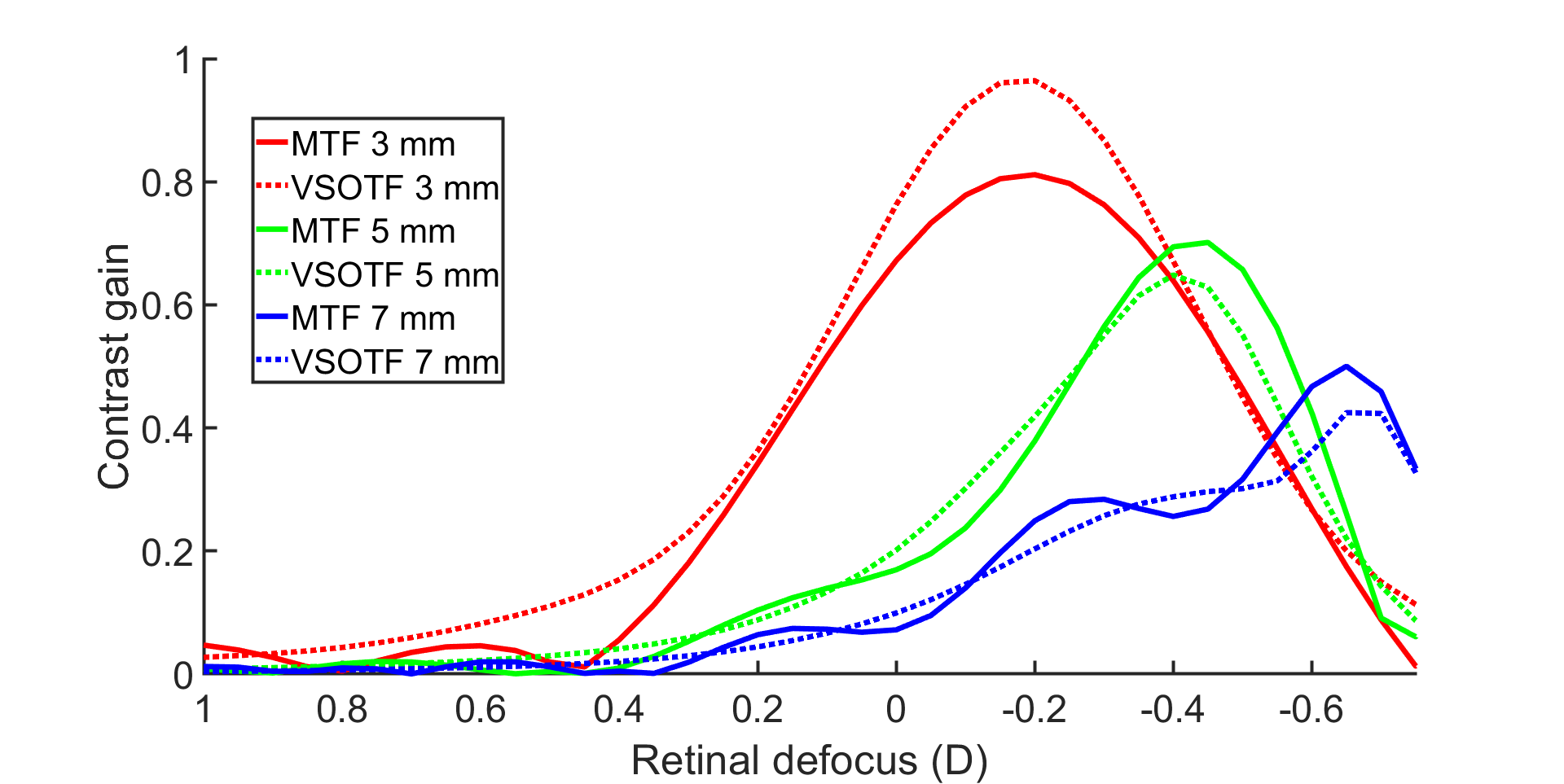

Supplement: Supplementary file 4 [file Image_3.tif]

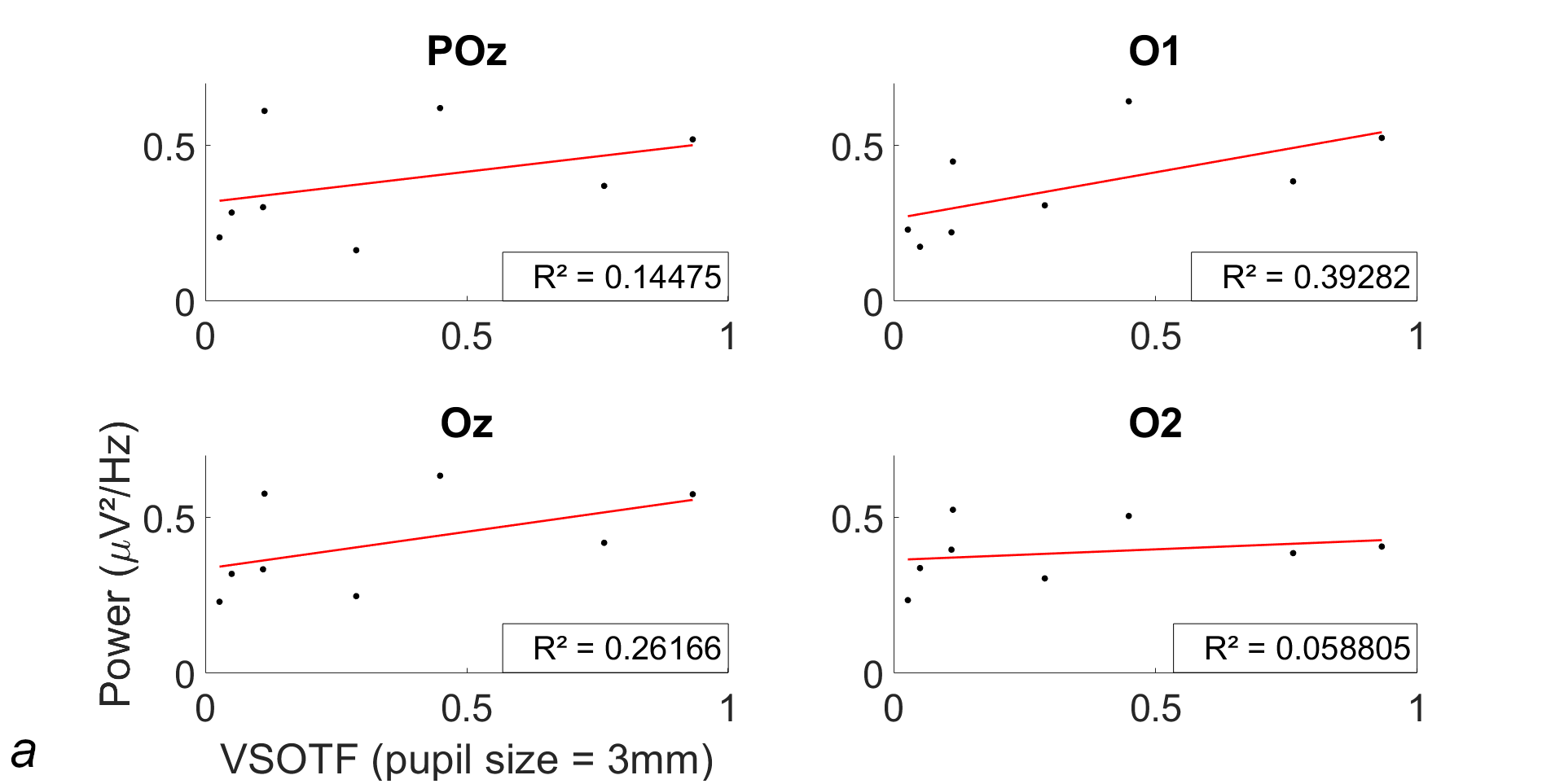

Supplement: Supplementary file 5 [file Image_4.tif]

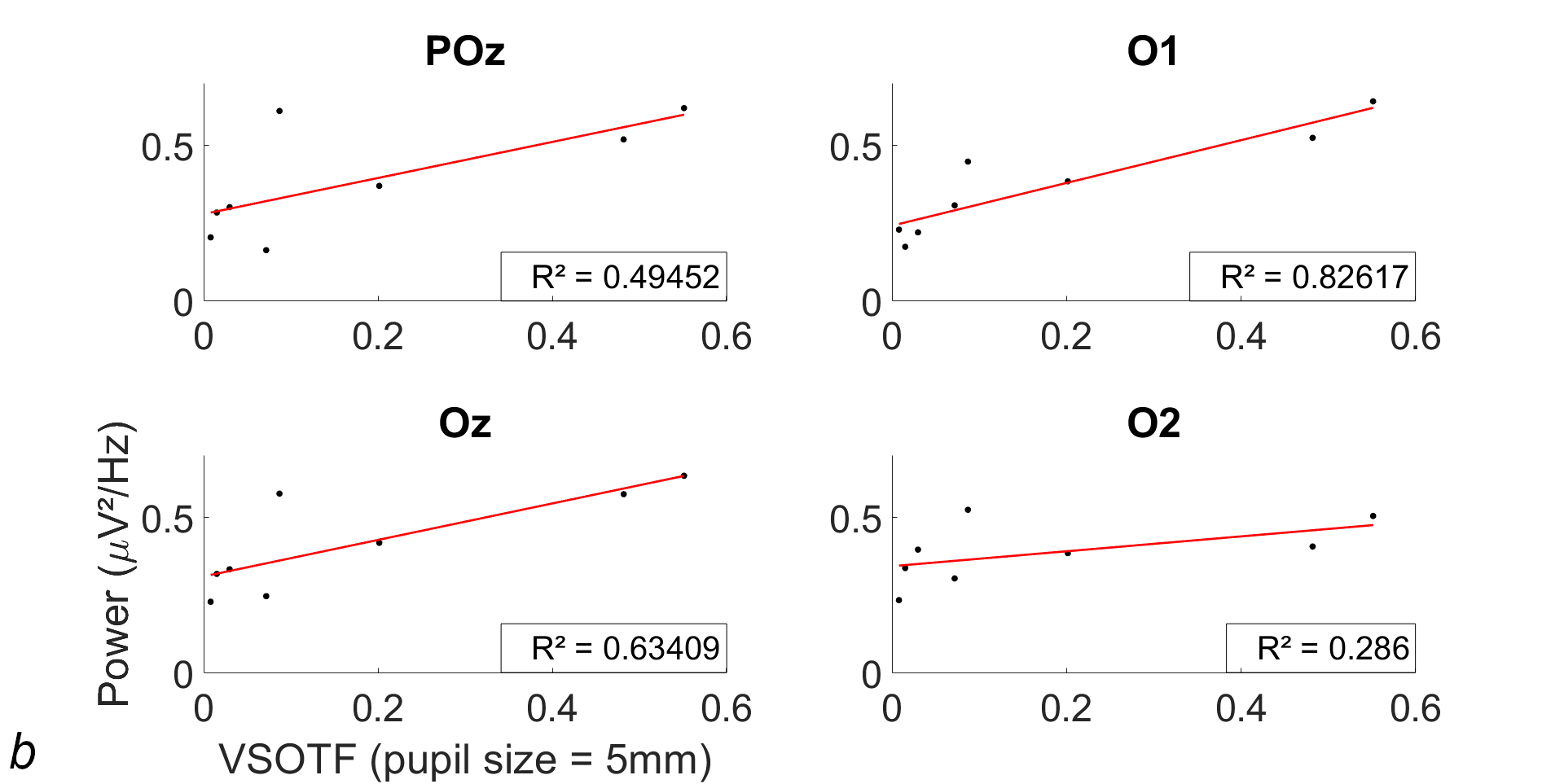

Supplement: Supplementary file 6 [file Image_5.tif]

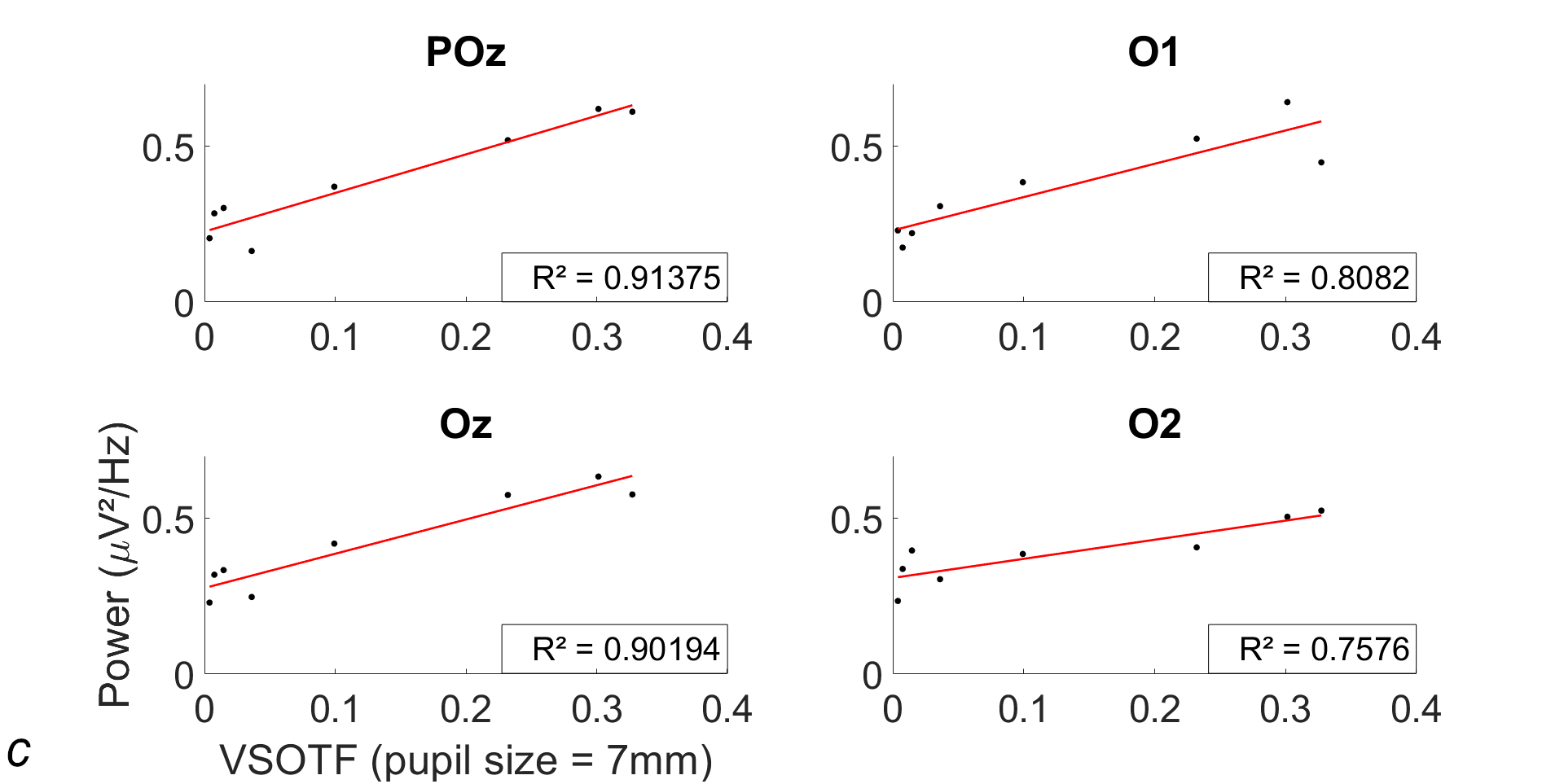

Supplement: Supplementary file 7 [file Image_6.tif]

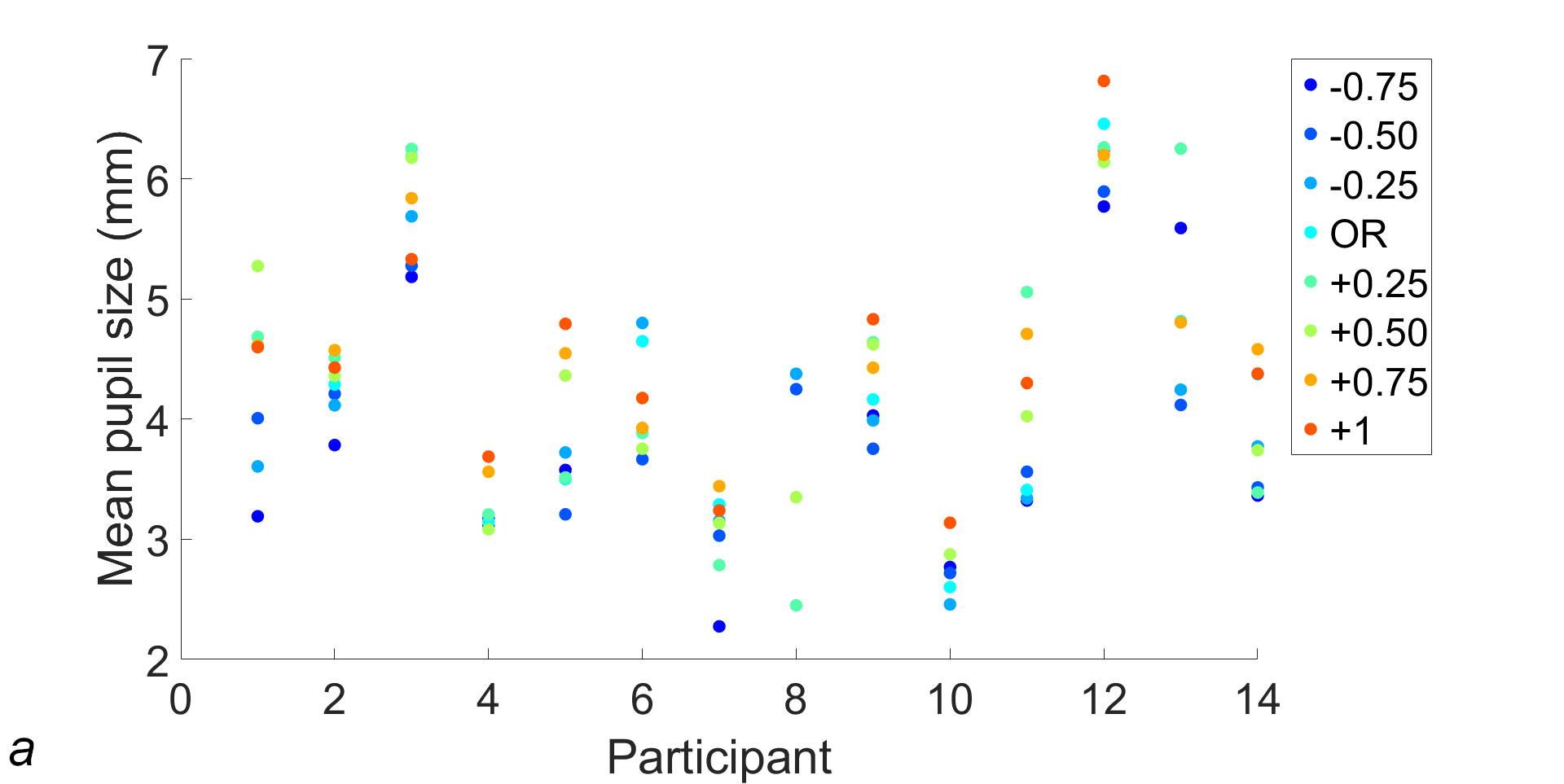

Supplement: Supplementary file 8 [file Image_7.tif]

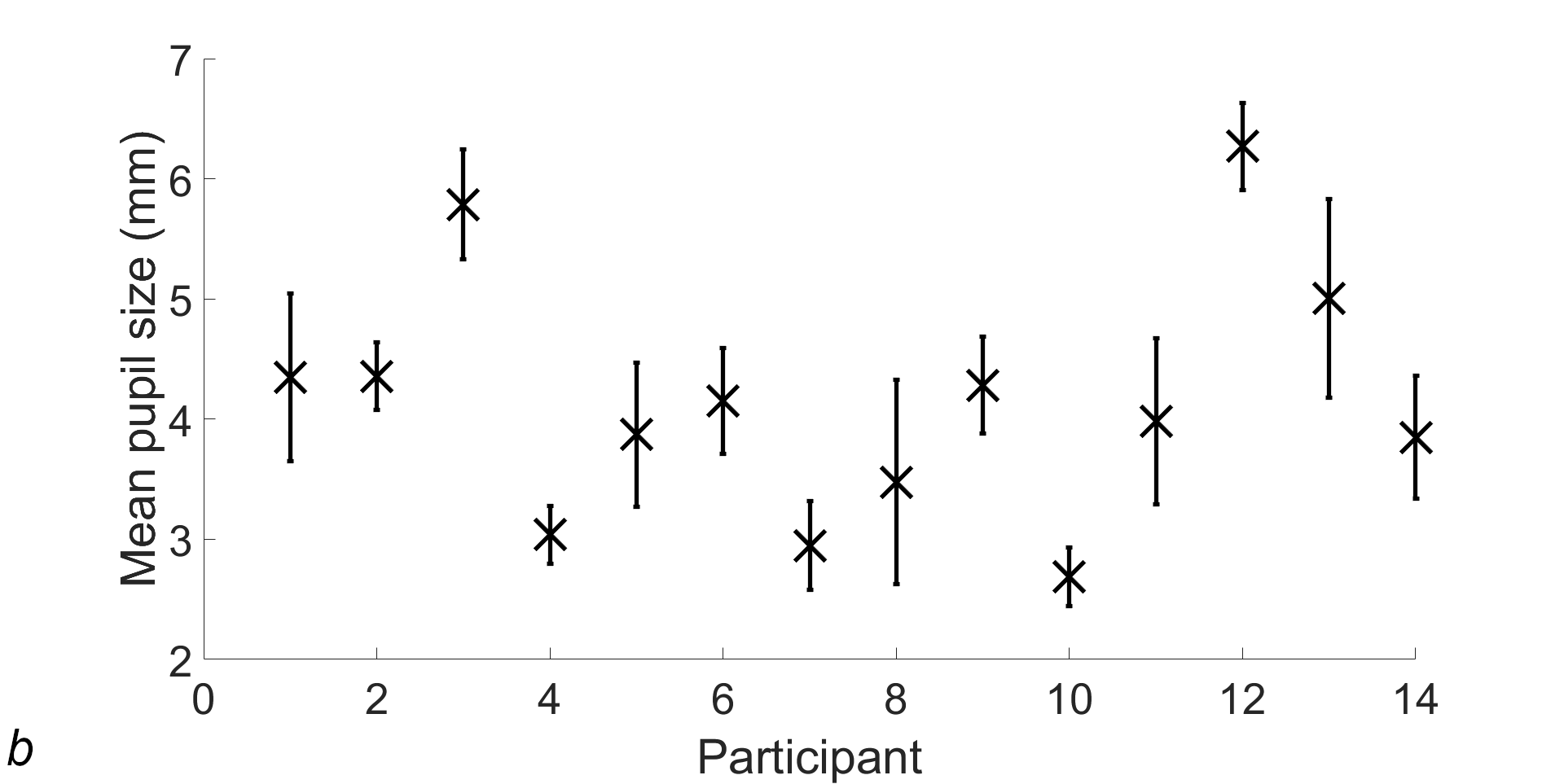

Supplement: Supplementary file 9 [file Image_8.tif]

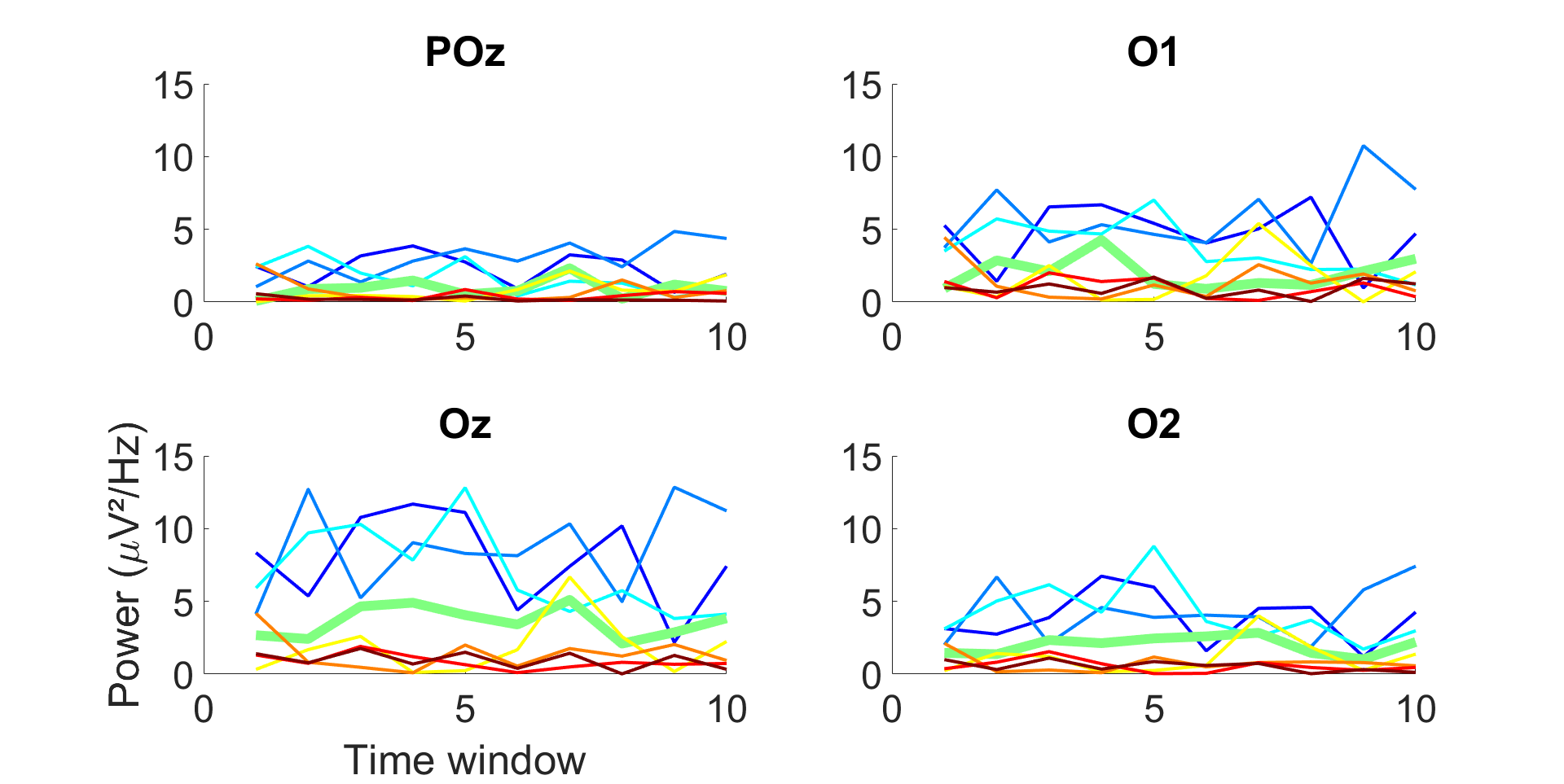

Supplement: Supplementary file 10 [file Image_9.tif]
